# Stability-Indicating HPLC Method for Simultaneous Determination of Chloramphenicol, Dexamethasone Sodium Phosphate and Tetrahydrozoline Hydrochloride in Ophthalmic Solution

**DOI:** 10.15171/apb.2016.020

**Published:** 2016-03-17

**Authors:** Hashem AlAani, Yasmin Alnukkary

**Affiliations:** ^1^ Department of Chemistry, Faculty of Science, Damascus University, Damascus, Syria.; ^2^ Department of Pharmaceutical Chemistry and Drug Quality Control, Faculty of Pharmacy, Damascus University, Damascus, Syria.

**Keywords:** Chloramphenicol, Dexamethasone Sodium Phosphate, Tetrahydrozoline Hydrochloride, HPLC, Ophthalmic Solution, Stability-indicating

## Abstract

***Purpose:*** A simple stability-indicating RP-HPLC assay method was developed and validated for quantitative determination of Chloramphenicol, Dexamethasone Sodium Phosphate and Tetrahydrozoline Hydrochloride in ophthalmic solution in the presence of 2-amino-1-(4-nitrophenyl)propane-1,3-diol, a degradation product of Chloramphenicol, and Dexamethasone, a degradation product of Dexamethasone Sodium Phosphate.

***Methods:*** Effective chromatographic separation was achieved using C18 column (250 mm, 4.6 mm i.d., 5 μm) with isocratic mobile phase consisting of acetonitrile - phosphate buffer (pH 4.0; 0.05 M) (30:70, v/v) at a flow rate of 1 mL/minute. The column temperature was maintained at 40°C and the detection wavelength was 230 nm.

***Results:*** The proposed HPLC procedure was statistically validated according to the ICH guideline, and was proved to be stability-indicating by resolution of the APIs from their forced degradation products.

***Conclusion:*** The developed method is suitable for the routine analysis as well as stability studies.

## Introduction

**Figure 1:**
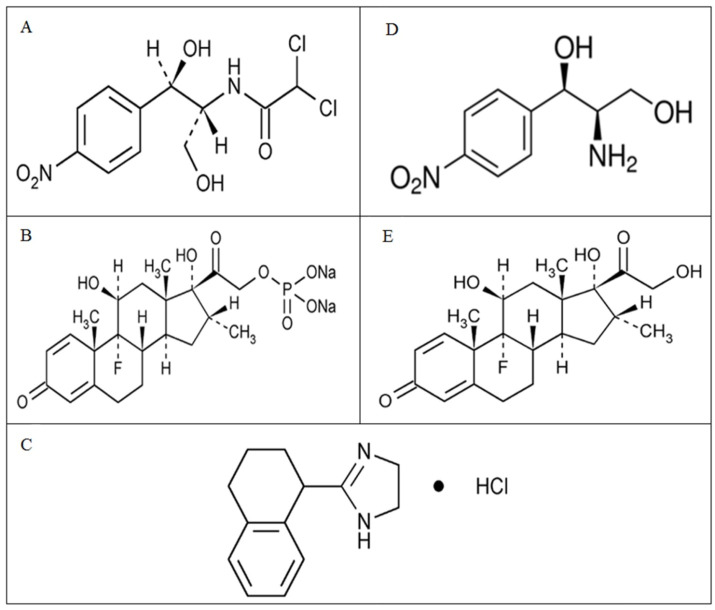
Chemical structures of (A) CAP (B) DSP (C) THC (D) AMPD (E) DEX

Chloramphenicol (CAP), Figure 1,A, is a bacteriostatic antibiotic.^1^ Dexamethasone Sodium Phosphate (DSP), Figure 1,B, is an inorganic ester of dexamethasone, that suppresses the inflammatory response to a variety of agents.^2^ Tetrahydrozoline Hydrochloride (THC), Figure 1,C, is an imidazoline-derivative sympathomimetic amine, which temporary relief of conjunctival congestion, itching, and minor irritation.^3^ An ophthalmic solution contains CAP 0.5%, DSP 0.1%, and THC 0.025% is available in the market. It is indicated for keratitis, conjunctivitis acute and chronic infectious, inflammation of the uvea anterior, scleritis, and sympathetic ophthalmia.^4^

2-amino-1-(4-nitrophenyl)propane-1,3-diol (AMPD), Figure 1,D, is the hydrolysis product of Chloramphenicol.^5^ British Pharmacopoeia states that it should be less than 8%, with respect to Chloramphenicol, in the ophthalmic solution.^6^

Dexamethasone (DEX), Figure 1,E, is the hydrolytic derivative of Dexamethasone Sodium Phosphate.^7^ The allowable maximum limit for Dexamethasone in the solution for Injection is 0.5%, with respect to Dexamethasone Sodium Phosphate.^6^

The literature survey revealed that few methods determined simultaneously Chloramphenicol and Dexamethasone Sodium Phosphate^8,9^ in the presence of Dexamethasone.^10^ Therefore, the aim of this work was to develop and validate a new simple stability-indicating HPLC method for simultaneous determination of Chloramphenicol, its hydrolysis derivative (AMPD), Dexamethasone sodium phosphate, its hydrolysis derivative (Dexamethasone) and Tetrahydrozoline in the ophthalmic solution.

## Materials and Methods

### Chemicals and solutions

CAP was purchased from CHEMO, Spain; DSP and DEX were purchased from SYMBIOTICA, Malaysia; THC was purchased from S.I.M.S, Italy; AMPD was purchased from British Pharmacopoeia Commission Laboratory; and excipients were kindly supplied by DIAMOND PHARMA, Syria.

Acetonitrile used was of HPLC grade. All the other reagents used were of analytical grade. Purified water was used for making the solutions.

### Chromatographic conditions

Separations were performed with a HPLC (LA Chrom ELITE, VWR Hitachi, Germany, equipped with L-2130 pump, L-2200 auto sampler, L-2300 column oven, and UV photo diode array detector L-2455). The out-put signal was monitored and processed using EZ Chrom ELITE software‏.

The column used was Thermo Hypersil C18 column (250 mm, 4.6 mm i.d., 5 μm). The isocratic mobile phase comprised of mixture of acetonitrile - potassium dihydrogen phosphate buffer (pH 4.0; 0.05 M) (30:70, v/v). The mobile phase was filtered through 0.45 μm membrane filter, degassed in ultrasonic bath and pumped from the respective solvent reservoir at a flow rate of 1 mL/minute. The column temperature was maintained at 40°C and the detection wavelength was 230 nm. The injection volume was 20 µL. The column was equilibrated for about 60 minutes prior to injection.

### Preparation of standard solution

CAP (5000 μg/mL), DSP (1000 μg/mL), THC (250 μg/mL) and AMPD (400 μg/mL) stock solutions were prepared in mobile phase.

DEX solution (250 μg/mL) was prepared in acetonitrile. Then, dilution was made with mobile phase to obtain DEX stock solution with concentration of (5 μg/mL).

2 mL of each of the stock solutions were transferred into a 25 mL volumetric flask and diluted with mobile phase. The concentrations obtained were 400, 80, 20, 32, 0.4 μg/mL for CAP, DSP, THC, AMPD and DEX, respectively.

The standard solution was filtered using a 0.45 μm filter.

### Method validation

The proposed HPLC method was validated according to ICH guideline.^11^

### Forced degradation studies

#### Stock solutions

CAP (4000 μg/mL), DSP (800 μg/mL) and THC (200 μg/mL) stock solutions were prepared in mobile phase.

#### Degradation studies

5 mL of each of the stock solutions were transferred into a 50 mL volumetric flask, in each study.

**For acidic hydrolysis,** 2 mL of 2 M HCl was added, and the volumetric flask was kept at 70°C for about 3 hours in water bath. Then the solution was allowed to attend ambient temperature, neutralized by 2 mL of 2 M NaOH, and the volume was made up with mobile phase.

**For alkaline hydrolysis,** 1 mL of 0.1 M NaOH was added, and the volumetric flask was kept at 70°C for about 60 minutes in water bath. Then the solution was allowed to attend ambient temperature, neutralized by 1 mL of 0.1 M HCl, and the volume was made up with mobile phase.

**For oxidative degradation,** 3 mL of 3% H_2_O_2_ was added, and the volumetric flask was kept at 70°C for about 3 hours in water bath. Then the solution was allowed to attend ambient temperature and the volume was made up with the mobile phase.

**For thermal degradation,** the volumetric flask was kept at 70°C for 3 hours in water bath. Then the solution was allowed to attend ambient temperature and the volume was made up with mobile phase.

**For photolytic degradation,** the volumetric flask was subjected to both of the cool white fluorescent and near ultraviolet lamp with a maximum energy emission at 365 nm for 30 minutes. Then the solution was allowed to attend ambient temperature and the volume was made up with mobile phase.

All solutions were filtered with a 0.45 μm filter and injected in stabilized chromatographic conditions.

## Results and Discussion

### Method validation

The results of system suitability test from five replicate injections of standard solution were within the acceptable limits as per FDA guideline.^12^

The chromatograms of standard solution and excipients solution showed the absence of interfering peaks at the retention times of analytes in the excipients chromatogram, which demonstrates specificity of the method.

Good linearity was obtained in the studied ranges, as the correlation coefficients of the peak area responses versus concentrations calibration curves were more than 0.999.

This method was found to be precise as the RSD% of assay values at three concentrations (50%, 100% and 200%) for repeatability and intermediate precision (performed by three analysts) were less than 2%.

Recovery % of the analytes at each of added concentration (50%, 100% and 200%) was within the range of 98% to 102%, indicating that the method is accurate.

The summary of validation parameters of the proposed method is tabulated in Table 1.

**Table 1:** Summary of validation parameters

**Parameter**		**AMPD**	**THC**	**DSP**	**CAP**	**DEX**
**System suitability**	**RSD% of area**	0.1	0.2	0.2	0.1	0.3
	**RSD% of** **retention time**	0	0.2	0.6	0.4	0.7
	**Tailing factor**	0.8	1.6	1.2	1.2	1.1
	**Resolution**	-	6.7	4.4	11.6	21.1
	**Theoretical plates**	9745	7521	9646	14337	16507
**Linearity**	**Range (µg/mL)**	100 – 800	20 – 160	5 – 40	8 – 200	0.1 – 50
**Precision**	**Repeatability, RSD%**	0.9	0.4	0.5	0.5	1.1
	**Intermediate precision, RSD%**	1.4	1.5	1.4	1.3	0.6
**Accuracy**	**Mean recovery %**	99.6	100.4	99.9	100.9	100.1
**Sensitivity**	**Detection limit, (µg/mL)**	0.06	0.03	0.04	0.04	0.02
	**Quantification limit, (µg/mL)**	0.21	0.11	0.13	0.13	0.07

The robustness was evaluated by making small changes in some method parameters including mobile phase composition (± 1%), mobile phase pH (± 0.1), flow rate (± 0.1 mL/minute), column temperature (± 2°C), wavelength (± 2 nm), and injection volume (± 10 μL); System suitability parameters were within the acceptable limits in all varied chromatographic conditions, indicating that the method is robust.

However, in extended robustness study to evaluate the effect of larger variation in the chromatographic conditions, the resolution between THC and DSP peaks was found to be susceptible to the acetonitrile percentage increasing, as it became about 1.9 when the percentage was 32%. Thus, it's recommended to suitably control the mobile phase composition to get the best resolution.

### Forced degradation studies

In the chromatograms resulted from all degradation studies, there was no interference between the tested drugs and the degradation products.

**Figure 2:**
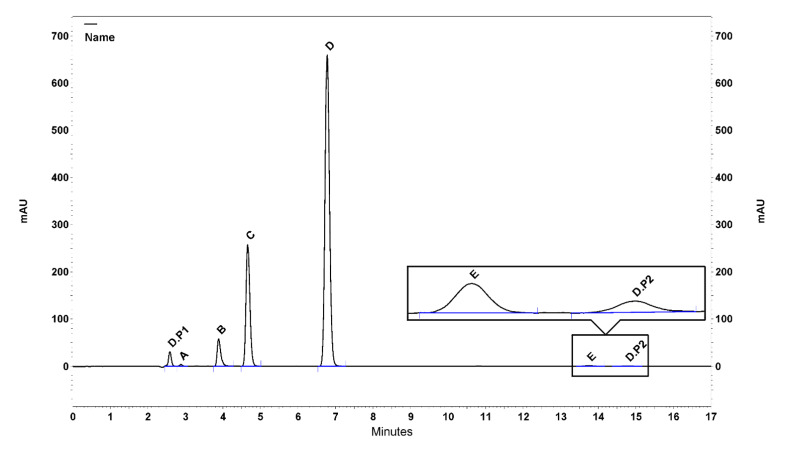
Chromatogram resulted from oxidative degradation study, in which (A) AMPD, (B) THC, (C) DSP, (D) CAP, (E) DEX, (D.P1) and (D.P2) Degradation products

The chromatogram resulted from oxidative degradation study is represented in (Figure 2), as an example.

The peak purity spectrum of each tested drugs was recorded using PDA detector. Peak purity results were greater than 0.99, which indicates that the peaks are homogeneous in all stress conditions tested and thus establishing the specificity and confirming the stability-indicating power of the developed method.^13,14^

Table 2 presents the forced degradation studies results.

**Table 2:** Forced degradation studies results

**Condition**	**THC**		**DSP**		**CAP**	
	**Assay**	**Peak purity**	**Assay**	**Peak purity**	**Assay**	**Peak purity**
**Acidic**	98.2%	1.000	96.9%	1.000	92.2%	1.000
**Alkaline**	97.2%	0.998	96.1%	1.000	97.4%	1.000
**Oxidative**	99.3%	1.000	96.9%	1.000	97.7%	1.000
**Thermal**	98.6%	1.000	97.5%	1.000	97.3%	1.000
**Photolytic**	97.1%	1.000	92.5%	1.000	94.8%	1.000

## Conclusion

A simple HPLC method has been developed and validated for the analysis of Chloramphenicol, Dexamethasone Sodium Phosphate and Tetrahydrozoline Hydrochloride in the presence of Dexamethasone and AMPD in ophthalmic solutions. The results of the stress testing revealed that the method is stability-indicating; therefore, this method can be used to analyze samples during stability studies and the routine assay. In addition, the method can be applied for the determination of Dexamethasone and AMPD in ophthalmic solutions.

## Ethical Issues

Not applicable.

## Conflict of Interest

The authors report no conflicts of interest.
